# Epigenetic Regulation and Gene Expression Profiles in Cervical Swabs: Toward Non-Invasive Biomarkers of Cervical Lesion Progression

**DOI:** 10.3390/epigenomes10010002

**Published:** 2026-01-07

**Authors:** Ivana Kašubová, Andrea Hornáková, Lucia Kotúľová, Tomáš Rokos, Zuzana Kolková, Andrea Kapinová, Terézia Pribulová, Erik Kozubík, Michal Kalman, Kamil Biringer, Erik Kúdela, Veronika Holubeková

**Affiliations:** 1 Biomedical Centre Martin, Jessenius Faculty of Medicine in Martin, Comenius University Bratislava, 03601 Martin, Slovakia; ivana.kasubova@uniba.sk (I.K.); andrea.hornakova@uniba.sk (A.H.); lucia.kotulova@uniba.sk (L.K.); zuzana.snahnicanova@uniba.sk (Z.K.); andrea.kapinova@uniba.sk (A.K.); 2 Clinic of Obstetrics and Gynaecology, Jessenius Faculty of Medicine in Martin, Comenius University Bratislava, 03601 Martin, Slovakia; rokos1@uniba.sk (T.R.); vlcakova8@uniba.sk (T.P.); erik.kozubik@uniba.sk (E.K.); kamil.biringer@uniba.sk (K.B.); erik.kudela@uniba.sk (E.K.); 3 Department of Pathological Anatomy, Jessenius Faculty of Medicine in Martin, Comenius University Bratislava, 03601 Martin, Slovakia; michal.kalman@uniba.sk

**Keywords:** methylation, gene expression, HPV, microenvironment

## Abstract

**Background/Objectives**: Cervical cancer is a common malignancy in women worldwide, closely associated with persistent human papillomavirus (HPV) infection. Epigenetic mechanisms, particularly promoter methylation, may contribute to tumour progression. This pilot study aimed to analyse the promoter methylation patterns and gene expression of selected genes (*DNMT*, *BCL2*, *CDH1*, *CD8A*, *MUC1*, *ALCAM*). The goal was to identify associations between promoter hypermethylation, gene expression, and HPV infection in cervical swab specimens obtained from patients with low-grade squamous intraepithelial lesions (SILs), high-grade SILs, or squamous cell carcinomas. **Methods**: A total of 81 cervical swab samples from Slovak participants were included in the study. DNA methylation and gene expression profiling was performed using real-time PCR (qPCR) and pyrosequencing. **Results**: *BCL2* expression was significantly reduced across all lesion grades. *CD8A* expression was slightly elevated in low- and high-grade SILs, particularly in HPV-positive samples. *MUC1* showed variability with lesion grade. No statistically significant differences in DNA methylation were observed across groups stratified by HPV status, community state type, and lesion grade. **Conclusions**: Our findings suggest that *BCL2* downregulation and gene activity variability influenced by the vaginal microbiome may play a role in cervical lesion progression. These results highlight potential non-invasive biomarkers for monitoring cervical lesions.

## 1. Introduction

Cervical cancer is the fourth most common malignancy in women worldwide [1] and is usually accompanied by precursor cervical lesions, cytologically classified as atypical squamous cells of undetermined significance (ASCUS), low-grade squamous intraepithelial lesions (LSIL), and high-grade squamous intraepithelial lesions (HSIL) [2,3]. Current guidelines for cervical cancer screening recommend investigation of morphological changes in cytology smear in time-defined intervals and human papillomavirus (HPV) testing in suspect cases. In long-term cervical lesions, an expert colposcopy and biopsy withdrawal are recommended. Histological evaluation of the biopsy classifies morphological changes into cervical intraepithelial neoplasia grades 1 to 3 (CIN1–3) and/or carcinoma in situ (CIS) [4]. CIN1 lesions frequently regress spontaneously, and a subset of CIN2/3 lesions may also regress under conservative management; the likelihood of regression depends on factors such as lesion grade, patient age, HPV status, and duration of follow-up [5]. Recent systematic reviews and meta-analyses indicate that regression rates vary across studies and patient populations, with higher regression observed in younger women and in lesions with favourable biological profiles, whereas progression is more likely in older women, persistent high-risk HPV infections, and high-grade lesions [6]. The incidence of cervical cancer is disproportionately higher in less developed countries, possibly due to poor access to screening, public health priorities, poor HPV vaccination, technology, healthcare infrastructure, lifestyle modifications, and other factors.

Etiologically, cervical cancer is closely associated with persistent infection by high-risk HPVs, particularly types 16 and 18 [7]. The oncogenic activity of HPV viruses is driven by the overexpression of E6 and E7 oncoproteins, which dysregulate the cell cycle, ultimately leading to malignant transformation [8]. These oncoproteins can also modulate epigenetic regulation: E6 and E7 have been shown to upregulate DNA methyltransferases, leading to hypermethylation of tumour suppressor gene promoters and global alterations in DNA methylation patterns, which contribute to cervical carcinogenesis [9]. LSIL patients are typically associated with HPV infection, which may resolve spontaneously, but approximately 10% of LSIL cases progress to pre-malignant HSIL within a 2-year follow-up [2,10]. In most patients, however, the host’s immune system can eliminate HPV infection, especially CIN1 and a subset of CIN2/3, and the components of the responses may be promising biomarkers [5].

Cervical cancer is a multi-aetiology disease, and HPV infection alone is not a sufficient cause of cervical cancer [11]. It has been found that abnormal gene methylation on cytosine residues plays a role in cell proliferation, apoptosis, cell cycle, and transformation during cervical carcinogenesis through abnormal gene expression. This epigenetic modification could serve as a biomarker for early detection, diagnosis, and prognosis in cancer [12,13]. Hypermethylation modification is positively correlated with the degree of cervical lesions; therefore, DNA methylation detection is a promising tool in some phases of cervical cancer diagnosis [14,15]. Given the high prevalence of HPV infection, exploring the potential relationship between HPV infection, host gene methylation, and HPV gene methylation is crucial for developing new strategies for prevention, diagnosis, and treatment [16].

In this work, we selected a panel of genes—*DNMT1*, *BCL2*, *CDH1*, *CD8A, MUC1*, and *ALCAM*—as they represent fundamental processes involved in HPV-induced carcinogenesis, including regulation of methylation (*DNMT*), apoptosis (*BCL2*), immune response (*CD8A*), adhesion/invasiveness (*ALCAM*, *CDH1*), and mucosal protection and epithelial differentiation (*MUC1*). Their methylation status varies across lesion stages and may indicate progression. The panel was designed as a research panel to assess the association between methylation, gene expression and the cervicovaginal microenvironment. Unlike clinically validated methylation markers used in CIN2+ triage (e.g., T lymphocyte maturation associated protein gene (*MAL*), cell adhesion molecule 1 (*CADM1*), paired box 1 (*PAX1*)), which were not included due to their diagnostic focus, the panel we used provides a broader functional view of possible interactions between HPV infection, epigenetic regulation, and microbial composition. This limitation, however, allows the analysis of pathways not offered by routine diagnostic panels. DNA methylation is mediated by DNA methyltransferase (*DNMT*) enzymes that add methyl groups specifically to cytosines in gene promoters, thus controlling their expression (gene activation or repression) [17]. Some studies have reported that cancer development and the presence of HPV infection influence *DNMT* activity, leading to differentially methylated gene regions that arise in genome-wide methylation-specific tumour signatures in the presence of chronic inflammation [18].

Chronic inflammation in HPV-infected lesions inhibits apoptosis, promoting cell survival, proliferation, and an abnormal immune response [19]. In this context, epithelial-to-mesenchymal transition (EMT) is favoured, with frequent methylation of the *CDH1* (cadherin 1) promoter, leading to loss of tissue integrity and cell–cell adhesion [20,21]. Other genes of adhesion, immune response, and apoptosis, such as *MUC1* (mucin 1), *CD8A* (T-cell surface glycoprotein CD8 alpha chain), and *BCL2* (B-cell lymphoma 2), are frequently affected by methylation, and different gene expression profiles are observed.

Increasing evidence suggests that vaginal microbiota composition, grouped into community state types (CSTs), can modulate local inflammatory signalling and oxidative stress, which in turn influence *DNMT* activity and contribute to aberrant DNA methylation patterns associated with HPV persistence and lesion progression. Five main CSTs have been described based on four *Lactobacillus* species. CST I was dominated by *L. crispatus*, CST II by *L. gasseri*, CST III by *L. iners*, and CST V by *L. jensenii* [22]. Only CST IV is characterised by low or no lactobacilli and high content of anaerobic bacteria.

Only a few studies have analysed DNA methylation and current RNA expression in cervical precancerous lesions and cancer. Therefore, we focused on identifying DNA methylation patterns and corresponding gene expression changes in the above-mentioned selected genes. This study was performed in cervical swab samples, which were obtained by a non-invasive procedure, in contrast to biopsy or tissue collection. Cervical swabs represent a heterogeneous mixture of malignant and non-malignant epithelial cells, immune cells, and surrounding microenvironmental elements that interact with each other and may contribute to cancer progression. The heterogeneous cellular composition of cervical swabs provides a realistic snapshot of the local microenvironment but also means that methylation and expression signals represent combined contributions from multiple cell types. Despite the limited number of samples, sufficient DNA and RNA quality is preserved for both epigenetic and gene expression analyses. We were also able to capture the local cervical microbiome by the method described earlier [23]. Our study’s aim was to determine whether specific methylation expression profiles derived from cervical swabs correspond to pathological stages of cervical disease and hold potential as minimally invasive biomarkers for improved clinical decision-making.

## 2. Results

### 2.1. Specimens and Clinicopathological Findings

Epigenetic analysis and gene expression profiling were performed on 81 cervical epithelial swab specimens. Analyses were performed on all collected samples. The average age of all study participants was 39.2 years (range, 19–77 years). The <50/≥50 age cut-off was chosen, based on established epidemiological and biological evidence, to reduce biological heterogeneity relevant to study outcomes. This choice is supported by studies demonstrating age-dependent variation in HPV prevalence and cervical lesion distribution, with a secondary increase around age 50 and older [24,25,26]. HPV infection with HPV16 or HPV18 genotypes was present in 42% (34/81) of cervical specimens, while other high-risk HPV types were detected in nearly 19% (15/81) of cervical samples. The study cohort consisted of 19% (15/81) negative for intraepithelial lesion or malignancy (NILM) controls, and 15% (12/81) ASCUS, 29.5% (24/81) LSIL, and 37% (30/81) HSIL samples. The overall characteristics of the study participants are presented in Table 1. HPV distribution significantly correlated with cytological findings. NILM was predominantly (94%) HPV-negative, while ASCUS, LSIL, and HSIL were predominantly positive. A detailed breakdown of HPV genotype distribution across cytological categories is shown in Table 2.

### 2.2. Methylation Analysis

Using pyrosequencing on the PyroMark Q48 Autoprep Instrument (Qiagen, Hilden, Germany), we detected the methylation status of the *DNMT*, *CD8A*, *MUC1*, *BCL2*, *CDH1*, and *ALCAM* genes. For result evaluation and statistical processing, the Box–Cox transformed Welch *t*-test was used. DNA methylation levels were compared across groups stratified by cytology grade, histology, HPV status, and cervicovaginal CST with predominance of *Lactobacillus iners* (CST III) and anaerobic bacteria (CST IV) compared to the control group (CST I, II, and V). Estimates represent effect sizes from regression models comparing mean methylation levels between groups. Thus, estimates close to zero indicate minimal average methylation differences. It is important to note that these effects sizes do not reflect the within-group variability of methylation. Fractional values (<1) indicate the proportion of methylated cytosines at analysed CpG sites in individual samples. No statistically significant correlations were detected between methylation and CST status (Table 3) or HPV 16/18 infection (Table 4). The small sizes of some subgroups (CST III and IV) may limit the statistical power of the analyses. Given the limited number of CpG sites analysed and the inherent cellular heterogeneity of cervical swab specimens, the sensitivity for detecting methylation differences is likely reduced, which may contribute to the mostly non-significant findings observed.

The epigenetic profiles of *DNMT1*, *CD8A* and *MUC1* remained stable across the cytology groups. Other genes, such as *BCL2* and *CDH1*, showed significant hypo- (*p* = 0.045) and hypermethylation (*p* = 0.042), respectively, but these differences were not significant after *p*-value adjustment. *ALCAM* gene hypermethylation was significant in ACSUS (*p* = 0.016) and LSIL (*p* = 0.042) cytological lesions (Table 5).

Among the studied groups, according to the histological results of cervical biopsy, *MUC1* showed the greatest within-group variability in methylation levels (i.e., the largest dispersion among individuals), although the between-group mean differences were small, resulting in regression estimates close to zero. There was a statistically significant difference between the CIN2 and CIN3 groups. The observed variability between histological grades supports the hypothesis that *MUC1* methylation may be associated with lesion progression (Table 6).

### 2.3. Gene Expression Profiling

**Figure 1:**
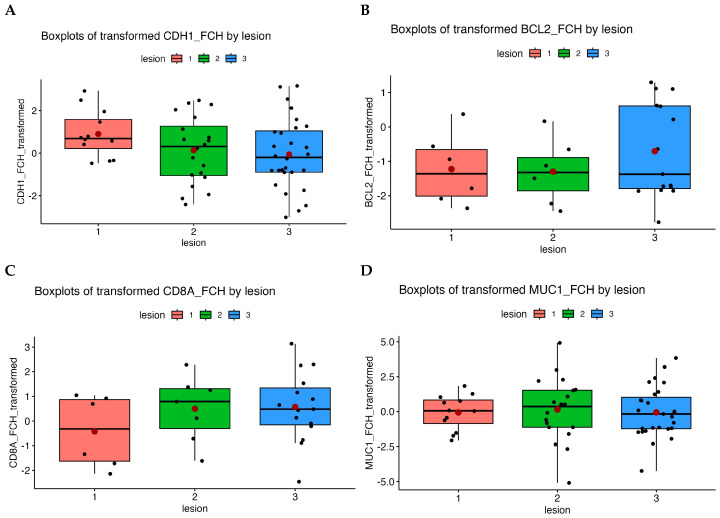
Boxplots of the transformed fold change (FCH) values according to the lesion grade. (**A**)—*CDH1*, (**B**)—*BCL2*, (**C**)—*CD8A*, and (**D**)—*MUC1*. Lesion grades: 1 = control (NILM/negative), 2 = low-grade lesions (ASCUS/LSIL), 3 = high-grade lesions (HSIL). Black line = median, red dot = mean.

Gene expression profiling was performed for selected genes—*CD8A*, *CDH1*, *MUC1*, and *BCL2*. Our results were evaluated according to HPV status, lesion grade, CST profile and participant age. We observed gene expression changes, expressed as fold change (FCH), depending on age and lesion type. FCH represents how many times gene expression changed compared with the controls (NILM lesions). FCH > 1 indicated increased expression, FCH < 1 indicated downregulation, and FCH = 1 indicated unchanged expression level. Changes in gene expression across lesion grades are shown in Figure 1, which presents boxplots of transformed FCH values for each gene according to lesion grade. For *CDH1* (Figure 1A) and *MUC1* (Figure 1D), expression values vary across lesion grades but without statistically significant differences, as evidenced by overlapping interquartile ranges and broad whiskers, indicating high sample variability. *BCL2* expression (Figure 1B) was significantly downregulated across all lesion grades compared with the controls (ASCUS FCH = 0.28, *p* = 0.00009; LSIL FCH = 0.27, *p* = 0.00004; HSIL FCH = 0.25, *p* = 0.0039) based on comparative testing with the control group. These results confirm reduced expression in lesions compared with controls, indicating decreased antiapoptotic activity of the *BCL2* gene. The expression profiles of the other analysed genes did not change significantly across lesion grades.

*CD8A* (Figure 1C) demonstrates a slight increase in expression in low- and high-grade lesions relative to controls, although these changes did not reach statistical significance, possibly due to sample size limitations and variability. In *CD8A*, expression was slightly increased in LSIL and HSIL lesions compared with the controls (FCH > 1). This might reflect increased immune activity (CD8^+^ T lymphocytes) with a higher lesion grade (*p* = 0.48). The lack of statistical significance may reflect high variability and small subgroup sizes. In HPV-positive specimens, the median fold change for *CD8A* was 2.48. Based on a linear model, our results suggest higher expression in HPV-positive lesions (*p* = 0.20), although differences were not statistically significant.

**Figure 2:**
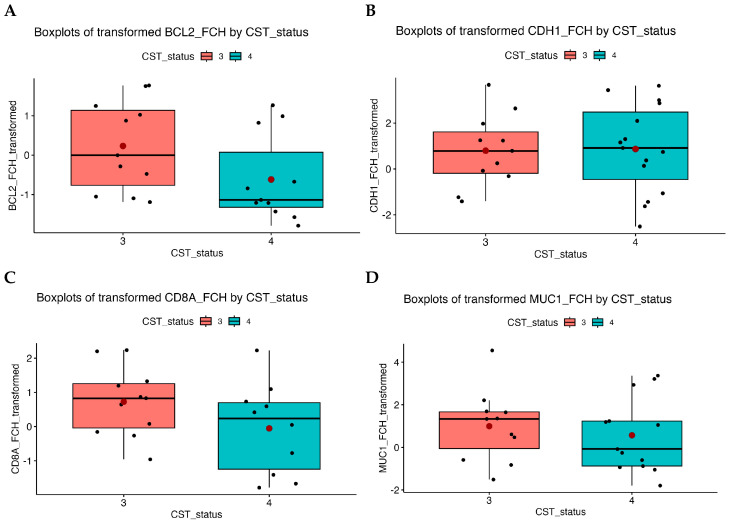
Boxplot of transformed FCH values according to CST status. (**A**)—FCH_BCL2, (**B**)—FCH_CDH1, (**C**)—FCH_CD8A, and (**D**)—FCH_MUC1. A lower median expression of *BCL2* was observed in CST IV compared with CST III, suggesting downregulation associated with CST IV microbial composition. Black line = median, red dot = mean.

Expression of *BCL2* was also significantly reduced in samples with a CST IV vaginal microbiome compared with the controls. Visualisations according to the CST profile are shown in Figure 2. Analyses accounting for HPV status showed no statistically significant differences, indicating that *BCL2* downregulation was largely independent of HPV infection, while CST IV samples consistently showed lower expression.

*CD8A* and *MUC1* expression showed some variability across CST profiles (Figure 2C,D), but no statistically significant differences were observed.

For the *CDH1* gene, we observed differences between CST III and CST IV when evaluating expression according to CST status. In CST III, expression was higher than in the control group (FCH = 2.08), whereas in CST IV it was similar to control levels (FCH = 0.94). However, these differences were not statistically significant (*p* = 0.33; *p* = 0.88) and therefore do not provide evidence for expression changes associated with CST status. Although *CDH1* expression appeared slightly lower in low- and high-grade lesions compared with controls, these differences were not statistically significant.

Interestingly, for the *CDH1* gene—unlike the other genes studied—we detected a slight correlation between age and gene expression (0.02 < *p* < 0.03). Older individuals showed higher *CDH1*_FCH values. Although this association was statistically significant, it accounted for only a small proportion of the total variability (R^2^ = 0.139 for the entire model). *CDH1* expression exhibited a weak correlation with age, suggesting minimal clinical relevance.

An exploratory analysis comparing DNA methylation levels with corresponding gene expression changes was performed for genes *CDH1*, *MUC1*, *CD8A*, and *BCL2* with available paired data. Across these genes, a trend toward an inverse association between methylation and expression was observed. However, due to the limited number of overlapping samples between methylation and expression datasets, these analyses lacked sufficient statistical power.

## 3. Discussion

The presented results are based on methylation analysis and gene expression profiling of cervical swab samples. Unlike most previous studies focusing on tissue samples, our study used a non-invasive collection method with clinical samples from Slovak participants. Cervical swabs are commonly used in cervical cancer screening and diagnostics as an initial, non-invasive screening tool for collecting cervical cells for laboratory examination. However, this non-invasive approach may also represent one of the study’s limitations. Cervical swab specimens contain a heterogeneous mixture of epithelial, stromal, and immune cells, along with mucus and microbial components, which may reduce the sensitivity of methylation and gene expression assays. This cellular heterogeneity may have contributed to the predominantly non-significant findings observed in our study.

The gene panel in this study was designed as a research-focused panel to assess the relationship between DNA methylation, gene expression, and the cervicovaginal microenvironment. This approach allowed us to explore how local microbial community composition and HPV infection influence host gene regulation and epigenetic patterns, providing mechanistic insights into lesion development and progression. *DNMT1* is central to DNA methylation maintenance and has been implicated in epigenetic dysregulation in HPV-related lesions. *CD8A* reflects local immune activity, which may influence lesion outcomes. *MUC1* and *CDH1* are involved in epithelial cell adhesion and integrity, processes that can be disrupted during dysplasia. *BCL2* regulates apoptosis, and its downregulation may facilitate cell survival in transforming lesions, while *ALCAM* plays a role in cell–cell interactions and tissue architecture. However, established methylation biomarkers for CIN2+ detection, such as *CADM1*, *MAL*, and *PAX1*, were not included. Incorporating these validated markers in future studies could complement our gene panel and increase clinical relevance for high-grade lesion detection.

A recent study highlighted DNA methylation as a potential biomarker for stratifying HPV-positive women [15]. Another study reported that DNA methylation in cervical swabs could serve as a diagnostic tool for ovarian cancer. They concluded that methylation differences were insufficient to distinguish between benign and malignant ovarian diseases, likely due to sample heterogeneity [27]. Our findings similarly indicate that cervical swabs have low sensitivity, likely underrepresenting abnormal cells. The methylation profiles of *CD8A*, *CDH1*, and *BCL2* remained stable across HPV status, CST profile, and lesion grade, suggesting a weaker signal compared with tissue samples.

Although these genes could serve as methylation markers, we observed no significant differences in our non-invasive samples with respect to HPV status or CST profile. In a previous study, *CDH1* promoter methylation was higher in CIN lesions and carcinomas than in controls, with no association with HPV status or stage, consistent with our findings [28].

Our results also showed variability in *MUC1* methylation depending on lesion status. Only a limited number of studies have investigated *MUC1* methylation in cervical lesion swabs or tissue samples. Although the observed variability did not reach statistical significance, it may reflect biological differences related to lesion progression. The absence of significant findings may be due to the small cohort size or sample heterogeneity. Nevertheless, the observed variability is consistent with the hypothesis that epigenetic alterations contribute to lesion progression.

Previous studies have shown that DNA methylation regulates *MUC1*, identifying it as an epigenetically controlled gene [29], and that promoter methylation of mucins, including *MUC1*, generally decreases across tumours [30]. In cervical lesions, our results suggest lesion-dependent variability in *MUC1* methylation and gene expression; however, most changes were not statistically significant. Validation in larger tissue sample cohorts is needed.

Recent advances in immunotherapy highlight the importance of T cells, yet knowledge of CD8⁺ T cell diversity and function in cervical cancer remains limited. CD8⁺ T lymphocytes play a key role in antitumor immunity [31,32]. Previous studies showed lower numbers in HPV-negative samples and increasing levels with CIN severity [33]. In our study, although the differences were not statistically significant (*p* = 0.48; *p* = 0.20), we observed variability in CD8⁺ T lymphocyte levels in relation to lesion progression and HPV infection. These findings, together with previous reports, are consistent with the proposed role of *CD8A* in the host immune response to HPV infection and lesion development.

Apoptosis, a genetically regulated biological process, maintains tissue homeostasis by eliminating damaged or harmful cells, and its dysregulation can contribute to cancer development [34]. The Bcl-2 protein family regulates apoptosis, with altered *BCL2* expression playing a role in cervical cancer progression. Generally, increased *BCL2* confers resistance to cell death and is linked to poor chemotherapy response and prognosis [35,36,37,38,39,40]. Literature on *BCL2* is conflicting: some studies report decreased expression in advanced or high-risk lesions, while others show upregulation in early-stage cervical cancer [41,42,43,44,45].

Dimitrakakis et al. (2000) found that *BCL2* expression increased with CIN grade but decreased in carcinomas compared to premalignant lesions, suggesting stage-specific variability [46]. In our study, *BCL2* expression was significantly reduced across all grades of cervical lesions compared with controls, indicating decreased anti-apoptotic activity in cervical swab samples. This reduction may reflect early disturbances in apoptotic regulation that could contribute to cellular susceptibility to transformation, with potential stage-specific roles for *BCL2* in cervical carcinogenesis.

*BCL2* expression was also lower in CST IV samples, which are associated with vaginal dysbiosis and inflammation [47,48,49]. Changes may reflect associations with microbial composition rather than causal effects, and they appeared independent of HPV infection, consistent with earlier findings showing no direct correlation between HPV and *BCL2* expression [50,51].

E-cadherin plays a critical role in maintaining epithelial tissue integrity through cell adhesion. Downregulation of E-cadherin, often caused by transcriptional repression of *CDH1*, disrupts cell adhesion and polarity, and is associated with tumour progression [28,52,53,54]. The cervical microbiome also plays an important role in lesion progression. CST-IV is characterised by microbial imbalance [28,55]. In our cervical swab analysis, differences in *CDH1* expression were observed between CST III and CST IV. Although these differences were not statistically significant (*p* = 0.33; *p* = 0.88), they reflect variability in expression across different microbial community states. These results are consistent with the notion that microbial composition may influence epithelial cell adhesion by regulating *CDH1* expression, but no definitive conclusions can be drawn. Previous studies have shown that cervical dysbiosis is associated with HPV infection and CIN development [56]. Increasing attention is being paid to the microbiome’s influence on cervical cancer, and current evidence suggests that microbial composition may affect lesion progression. However, a clear cause-and-effect relationship has not yet been established, and the underlying mechanisms remain unclear [57].

Overall, our findings indicate that epigenetic alterations, HPV infection, gene expression changes, and the cervical microbiome may be interconnected in cervical lesion development. However, our study’s main limitations include the small cohort size, reliance on heterogeneous swab-based samples, and the absence of established CIN2+ methylation markers. Future research should therefore integrate tissue-level validation, larger participant groups, and expanded biomarker panels to improve diagnostic accuracy and confirm or refute these preliminary findings.

## 4. Materials and Methods

### 4.1. Participants and Clinicopathological Findings

Cervical specimens were collected from 81 subjects who underwent treatment at the Clinic of Obstetrics and Gynaecology, University Hospital in Martin. All participants provided written informed consent to participation in the study. The study protocol was approved by the Ethics Committee of the Jessenius Faculty of Medicine in Martin, Comenius University in Bratislava (Nos. EK-85/2020 and EK-05/2024). Participants were recruited consecutively. Inclusion criteria were age ≥ 18 years, available cytology and/or colposcopy, and sufficient swab material. Exclusion criteria were pregnancy, previous cancer treatment, and inadequate sample cellularity. Cytological samples were classified according to the 2001 Bethesda System for reporting cervical cytology [58]. Clinical diagnoses were confirmed by histopathological examination at the Department of Pathological Anatomy, Jessenius Faculty of Medicine in Martin, Comenius University in Bratislava and the University Hospital in Martin. The clinical and pathological data of all enrolled participants are summarised in Table 2.

### 4.2. Nucleic Acid Extraction

Genomic DNA was extracted from cervical cell samples using the MasterPure™ Complete DNA and RNA Purification Kit (Biosearch Technologies, Hoddesdon, UK) according to the manufacturer’s instructions. DNA was treated with RNase to eliminate RNA contamination. RNA was extracted using a miRNeasy Micro Kit (Qiagen, Hilden, Germany), and the quality of nucleic acids (DNA and RNA) was evaluated by fluorometry (Qubit 3.0 Fluorometer, Thermo Fisher Scientific, Waltham, MA, USA). Extracted nucleic acids were stored at −80 °C until analysis. Samples with sufficient concentration of DNA and RNA were analysed in this study.

### 4.3. Bisulphite Conversion and Quantitative Pyrosequencing

For subsequent methylation analysis by pyrosequencing, 0.5 µg of isolated DNA was subjected to bisulphite conversion using the EpiTect Bisulfite Kit (Qiagen, Hilden, Germany) according to the manufacturer’s instructions. Twenty nanograms of bisulphite-converted DNA was amplified using the PyroMark PCR Kit (Qiagen, Hilden, Germany). PCR primers and sequencing primers (Qiagen, Hilden, Germany) were designed to amplify and sequence selected CpG sites in the target genes in commercial assays (Hs_BCL2_01_PM analysing 3 CpG sites (PM00071295), Hs_CD8A_01_PM analysing 7 CpG sites (PM00010892), Hs_MUC1_01_PM analysing 4 CpG sites (PM00005964), Hs_ALCAM_03_PM analysing 4 CpG sites (PM00108717), and Hs_DNMT1_01_PM analysing 5 CpG sites (PM00075761), Qiagen, Hilden, Germany), and previously published CDH1 primers [28] that analyse 12 CpG sites. Pyrosequencing was carried out on the PyroMark Q48 Autoprep Instrument (Qiagen, Hilden, Germany) using PyroMark Q48 Advanced Reagents according to the manufacturer’s protocol. Each assay contained 4 pmol of the corresponding sequencing primer. Target CpG sites were analysed using PyroMark Q48 Autoprep Instrument Software (version 4.4.3; Qiagen, Hilden, Germany). The percentage of methylation at each CpG site was calculated as the C/T peak height ratio. Both fully methylated and unmethylated DNA controls (Qiagen, Hilden, Germany) were included in each run to ensure assay accuracy. PCR products were checked for specificity by gel electrophoresis prior to pyrosequencing. Pyrosequencing reactions were performed once per sample, without technical duplicates or triplicates.

### 4.4. Gene Expression Analysis

The effect of DNA hypermethylation on gene expression was evaluated by relative quantification. For gene expression analysis, 500 ng of RNA was reverse-transcribed to complementary DNA (cDNA) using the High-Capacity cDNA Reverse Transcription Kit (Applied Biosystems; Thermo Fisher Scientific, Waltham, MA, USA) in a final volume of 20 μL. Multiplex quantitative PCR reactions were performed in triplicate using 50 ng of cDNA, TaqMan^®^ Gene Expression Master Mix (Thermo Fisher Scientific, Waltham, MA, USA), and Taq-Man gene expression assays (BCL2, Hs00153350_m1; CD8A, Hs00233520_m1; MUC1, Hs00904327_m1; GUSB, Hs99999908_m1; CDH1, Hs01013959_m1; ACTB, Hs99999903_m1; and GAPDH, Hs99999905_m1; Thermo Fisher Scientific, Waltham, MA, USA). Relative quantification was run in QuantStudio 5 (Applied-Biosystems; Thermo Fisher Scientific, Waltham, MA, USA) with amplification efficiencies above 90% for all assays. The fold change was calculated using the standard formula (2^−ΔΔCt^) and glyceraldehyde-3-phosphate dehydrogenase (*GAPDH*), beta-actin (*ACTB*), and finally glucuronidase (*GUSB*) were selected as reference genes due to their previously reported stable expression in cervical epithelial cells and swab specimens [59,60,61].

### 4.5. Statistical Analysis

Statistical analysis was performed on 81 cervical swabs with available clinicopathological data that underwent pyrosequencing and quantitative PCR analysis. The R programming language (version 4.4.2) was used for all statistical analyses, along with the libraries cited in the References section [62,63,64,65,66,67,68,69,70,71,72,73].

Each gene was analysed separately. First, exploratory data analysis was performed, summarising medians and lower and upper quartiles for continuous variables and counts and percentages for factors. For the evaluation of methylation results and statistical processing, we used the Box–Cox transformed Welch *t*-test. Boxplots were used to visualise the distribution of methylation and FCH across factor levels. To approximate normality, an appropriate transformation of the response (FCH) was selected using the powerTransform function. Because multiple statistical tests were performed, *p*-values were adjusted using the Benjamini–Hochberg procedure to control the false discovery rate. As part of the fold-change analysis, a linear model was fitted with age and each factor variable as predictors. Model diagnostics were performed, followed by post hoc analyses in which estimated marginal means were computed and contrasted with the reference level (controls), and pairwise comparisons were conducted between levels of each factor. *p*-values less than 0.05 were considered statistically significant.

## 5. Conclusions

This study is the first to integrate DNA methylation and gene expression analyses in cervical swab samples from the Slovak population with confirmed precancerous or cancerous lesions. Although no statistically significant differences in DNA methylation patterns were detected, variability in *MUC1* methylation was observed, suggesting potential involvement in lesion progression. *BCL2* expression was significantly downregulated across all cervical lesion grades, and lower expression was also detected in samples characterised by a CST IV microbiome composition. Overall, these findings reflect the complex interplay between epigenetic regulation, gene expression, and the cervicovaginal environment in cervical epithelial cells. While these observations provide exploratory insights into the mechanisms of lesion development and progression, further studies with larger cohorts and tissue-based analyses are needed to validate these findings and clarify their biological relevance.

## Figures and Tables

**Table 1:** Clinicopathological characteristics of participants enrolled in the study (*n* = 81).

Variable	Category	*n*	%
Age	≤49.9	68	84%
	≥50	13	16%
HPV status (overall)	HPV16/18	34	42%
	Other hrHPV	15	18.5%
	HPV-negative	32	39.5%
Cytology	NILM	17	21.0%
	ASCUS	11	13.6%
	LSIL	24	29.6%
	HSIL	30	37.0%
IHC p16	Negative	3	3.7%
	Positive	48	59.3%
	Unknown	30	37%
IHC Ki67	Positive	46	57%
	Unknown	35	43%

NILM—negative for intraepithelial lesion or malignancy; LSIL—low-grade squamous intraepithelial lesion; HSIL—high-grade squamous intraepithelial lesion; ASCUS—atypical squamous cells of undetermined significance; IHC p16—marker of deregulated expression of p16INK4a, typically positive in transforming HPV infection; IHC Ki67—proliferation marker. The total numbers of HPV16/18, other high-risk HPV, and HPV-negative cases reflect all tested samples. Cytology-stratified counts may be lower because a few samples lacked cytology results or were unsatisfactory for evaluation.

**Table 2:** HPV distribution within cytological categories.

Cytology	HPV16/18	Other hrHPV	HPV-Negative
NILM	0 (0%)	1 (5.9%)	16 (94.1%)
ASC-US	6 (54.5%)	4 (36.4%)	1 (9.1%)
LSIL	13 (54.2%)	8 (33.3%)	3 (12.5%)
HSIL	12 (40%)	14 (46.7%)	4 (13.3%)

NILM—negative for intraepithelial lesion or malignancy; LSIL—low-grade squamous intraepithelial lesion; HSIL—high-grade squamous intraepithelial lesion; ASCUS—atypical squamous cells of undetermined significance. Values represent absolute numbers with percentages indicating the proportion of each HPV subgroup within the corresponding cytological category.

**Table 3:** Methylation levels in groups based on CST status, expressed relative to the control group (CST I, II, V; *n* = 14).

	Class III (*n* = 13)		Class IV (*n* = 20)	
	Estimate	*p*-Value	Estimate	*p*-Value
*DNMT1*	−2.226	0.714	2.895	0.596
*CD8A*	−0.192	0.417	−0.103	0.624
*MUC1*	0.001	0.957	−0.009	0.398
*BCL2*	−0.874	0.829	0.091	0.977
*CDH1*	−4.059	0.619	2.982	0.704
*ALCAM*	4.234	0.620	14.795	0.067

Abbreviations: ***n***, number of samples in group; CST, community state type.

**Table 4:** Methylation level in the HPV 16/18-positive group, expressed relative to the HPV 16/18-negative group (*n* = 47).

	HPV16/18 Positive (*n* = 34)	
	Estimate	*p*-Value
*DNMT1*	0.425	0.912
*CD8A*	−0.012	0.937
*MUC1*	0.001	0.755
*BCL2*	−0.213	0.922
*CDH1*	1.61	0.86
*ALCAM*	0.573	0.861

Abbreviations: *n*, number of samples in group; HPV, human papillomavirus.

**Table 5:** Methylation levels of selected genes in cytology lesions compared with the control group (NILM, *n* = 15).

	ASCUS (*n* = 12)		LSIL (*n* = 24)		HSIL (*n* = 30)	
	Estimate	*p*-Value	Estimate	*p*-Value	Estimate	*p*-Value
*DNMT1*	0.808	0.894	−0.551	0.913	−6.802	0.174
*CD8A*	−0.322	0.232	−0.075	0.743	−0.346	0.143
*MUC1*	−0.006	0.364	−0.002	0.680	−0.007	0.161
*BCL2*	4.555	0.118	5.843	0.045	3.025	0.295
*CDH1*	−10.360	0.230	−7.349	0.409	−19.990	0.042
*ALCAM*	9.685	0.016	7.189	0.042	0.339	0.925

Abbreviations: *n*, number of samples in group; NILM, negative for intraepithelial lesion or malignancy; LSIL, low-grade squamous intraepithelial lesion; HSIL, high-grade squamous intraepithelial lesion; ASCUS, atypical squamous cells of undetermined significance.

**Table 6:** Methylation levels in biopsy groups, expressed relative to the negative group (*n* = 6).

	CIN1 (*n* = 4)		CIN2 (*n* = 15)		CIN3 (*n* = 3)		CIN3/CIS (*n* = 27)		CA (*n* = 7)	
	Estimate	*p*-Value	Estimate	*p*-Value	Estimate	*p*-Value	Estimate	*p*-Value	Estimate	*p*-Value
*DNMT1*	−3.819	0.444	0.251	0.949	−4.505	0.733	2.122	0.589	−2.651	0.569
*CD8A*	−1.689	0.106	−1.257	0.114	−2.004	0.068	−0.863	0.247	−1.039	0.222
*MUC1*	0.006	0.08	−0.01	0.022	0.006	0.013	0.001	0.68	−0.002	0.768
*BCL2*	−9.128	0.268	−3.146	0.449	−10.466	0.412	1.232	0.761	−3.968	0.488
*CDH1*	−3.140	0.783	−1.433	0.827	−24.988	0.281	2.286	0.657	−14.979	0.058
*ALCAM*	−15.024	0.156	−5.697	0.155	−18.653	0.161	6.273	0.120	−10.850	0.069

Abbreviations: *n*, number of samples in group; CIN, cervical intraepithelial neoplasia; CIS, carcinoma in situ; CA, adenocarcinoma, or squamous cell carcinoma.

## Data Availability

The data is available upon request from the authors.
